# Scaled 3D-printed models of insect outer-ear with tympanic membranes and acoustic trachea preserving key acoustic features

**DOI:** 10.1016/j.csbj.2025.12.003

**Published:** 2025-12-09

**Authors:** Md Niamul Islam, Fabio A. Sarria-S, Fernando Montealegre-Z

**Affiliations:** School of Natural Sciences, University of Lincoln, Lincoln, Lincolnshire, United Kingdom

**Keywords:** Bioacoustics, Biomimetic replication, Bush-crickets, Deep segmentation, Laser-Doppler vibrometer, Micro-computed tomography

## Abstract

Katydids (Insecta, Orthoptera, Tettigoniidae) possess a sophisticated foreleg ear with two tympana receiving sound externally through a pinna that acts as a bat detector and internally through an acoustic trachea for conspecific communication. Their miniature scale hinders experimentation, prompting the use of scaled models, yet previous studies have not replicated the full pinna–tympana assembly or the complete acoustic trachea. In this study, micro-CT imaging, AI-assisted segmentation and multi-material 3D-printed assembly were used to generate scaled models. Scaled copies of the pinna-tympanum assembly and the complete acoustic trachea of the neotropical katydid *Copiphora gorgonensis* were fabricated from high-fidelity reconstructions. Flexible TPU membranes reproduced the expected pressure-driven vibration pattern at the scaled frequency, and when paired with rigid PLA pinnae, they captured the overall outer-ear acoustic response, producing ultrasonic gain within 70–110 kHz that is consistent with the *in vivo* bat-detection band. Separately, the pressure mapping of the scaled acoustic trachea confirms the spiracle as a filter and the exponential canal as a 17–21 dB amplifier, in line with simulations, consistent with the 1.3-cycle phase shift observed at 23 kHz in living insects. These matching results support the use of scaled biomimetic replicas as reusable, 3Rs-aligned partial substitutes and complements to live-insect acoustic studies in search of bio-inspired applications. Taken together, these outcomes provide a basis for translating the underlying acoustic and structural principles into future miniature engineering systems, including bio-inspired MEMS microphones and compact directional sensors, where passive amplification and filtering remain desirable for improving energy efficiency and signal clarity.

## Highlights

• Scaled 3D printed replica of katydid outer ear with tympana and acoustic trachea.
• TPU membranes with PLA pinnae reproduced resonance at ultrasonic bat calls.
• Trachea filtered and amplified certain frequency, matching live insect phase delay.
• Scalable non-destructive 3Rs-aligned methods alternate to small scale tests.
• Basis for fossilised insect acoustic analysis and bio-inspired miniature sensors.

## Graphical Abstract

**Figure:**
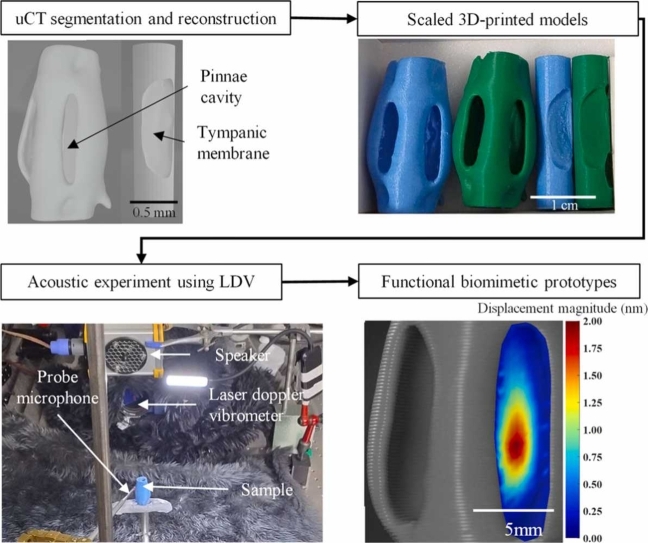

## 1 Introduction

Acoustic localisation is a fundamental survival trait: predators must pinpoint prey, prey must track predators, and both must identify conspecifics [1]. Most vertebrates solve this task with binaural cues — inter-aural time differences (ITDs) and inter-aural level differences (ILDs) [2]. For miniature animals, however, head width quickly approaches a fraction of the relevant wavelength, compressing ITDs to microseconds and ILDs to fractions of a decibel [3]. Katydids (or bush-crickets: Orthoptera, Tettigoniidae), a clade exceeding 8300 described species [4], overcome this scaling problem by relocating their ears to the forelegs. Separating the pair of ears by the width of the body, rather than the head, enlarges path‑length disparity and restores usable phase lags even for ultrasonic signals emitted in courtship or predation [5], [6].

Katydids remain unique among invertebrates for developing an auditory biophysical mechanism analogous to that of mammals [7], [8]. Each ear contains two thin tympana situated proximal on the foretibia and supplied by dual acoustic routes. External sound acts directly on the membrane surface, whereas internal sound enters a thoracic spiracle, propagates through an exponential acoustic trachea and impinges upon the inner membrane face [6], [9]. In *Copiphora gorgonensis*, a model species of insect hearing, the exponential horn‑shaped trachea boosts pressure by ∼15 dB and slows the phase by approximately 25 % relative to the external path, generating a pressure‑difference receiver [9]. More than 65 % of katydids add a third refinement: cuticular pinnae fold over the tympana, creating Helmholtz‑like cavities that deliver an additional 20–30 dB gains above 60 kHz, matching the echolocation band of gleaning bats [10], [11]. A fluid‑filled cavity covering the *crista acustica* enhances dispersion of travelling‑wave for frequency analysis, mirroring the tonotopic processing of the vertebrate cochlea [12].

Direct experimental work at the micro-scale of the katydid ear presents a significant challenge. The tympanic membranes are delicate, while the acoustic trachea is buried deep within the tibia [13]. Thus, non-invasive techniques are employed, such as laser-Doppler vibrometry (LDV) of intact specimens [14] and finite‑element models built from µCT volumes [15], [16], [17], which also limits the functional insights of ear mechanics. However, a better alternative is experimenting on scaled replicas fabricated via 3D printing (additive manufacturing). This valuable manufacturing process is capable of accurately replicating the complex anatomy of insect auditory structures. Scaled replicas are effective for acoustic experiments because sound–structure interactions depend primarily on the ratio between structural dimensions and the acoustic wavelength, rather than on their absolute size. For example, if the ear model is enlarged ten times (10 ×) and tested at frequencies ten times lower (1/10 ×), the relevant dimensionless relationships are maintained. However, while geometric scaling preserves these wavelength-to-size ratios, it does not achieve complete replication of all physical processes. At lower drive frequencies, the viscous and thermal boundary layers become proportionally thicker relative to the cavity dimensions, although at the present scale they occupy only a small fraction of each cross-section and predominantly act to smooth gain and phase responses rather than shift the principal resonance bands. Consequently, the propagation of sound, the development of resonances and the overall vibration patterns occur in broadly the same relative manner as in the natural ear, even if certain fine-scale mechanical effects are not reproduced exactly. Based on this phenomenon, previous studies have used 3D printing to replicate the function of the pinnae [10] and produce simplified insect ear models with flexible membrane material [18].

However, the precise fabrication of a fully functional katydid outer ear, flexible tympanic membranes with the rigid cuticle and pinnae, remains a significant challenge, as demonstrated in recent multimodal additive manufacturing of tympanic membranes replicating their acousto-mechanical performance [19]. The complex structural geometry and differences in the stiffness of printing materials, adhesion limitations, and segmentation accuracy pose additional obstacles to creating biologically accurate scaled models [20]. Despite these challenges, achieving a fully functional 3D-printed outer ear and acoustic trachea offers considerable advantages, as it enables scalability for biophysical measurements and controlled manipulation of structural parameters such as horn flare, cavity volume, and membrane stiffness. Working with scaled-up replicas not only simplifies experimental procedures and visualisation but also allows for future systematic testing of design variations to uncover the principles of frequency filtering and mechanical coupling [15]. These models provide a powerful platform for validating numerical simulations, optimising structural performance, and guiding the development of biomimetic auditory systems [16].

In this study, we present the first scaled, 3D-printed, multi-material functional replica of the outer ear of the neotropical katydid *C. gorgonensis*, integrating flexible tympana with the rigid pinnae and the complete spiracle-to-tympanum acoustic trachea. Built from high-resolution µCT data, AI-assisted segmentation and advanced 3D-printing techniques, the model reproduces the full geometry of the native outer ear and acoustic tracheal system. Unlike previous work, which characterised pinna mechanics without tympanic membranes [10], produced simplified tympanal membranes without the enclosing pinna cavity [18], [19], or examined the acoustic trachea only through finite-element simulations [16], the present study provides the first complex physical reconstruction of the complete katydid outer-ear and tracheal pathway. This enables experimental assessment of key acoustic features, including pinna-induced gain, tympanal vibration and tracheal sound transmission, allowing direct comparison between scaled models and in vivo results. The promising bioacoustics response of the scaled models establishes a platform for systematic investigation of miniature filters, amplifiers and pressure‑difference receivers, providing a potential pathway for translating insect biomechanics into future miniature acoustic sensors. As additive manufacturing technologies continue to advance, with increasing printing resolution and multi-material precision, the same design principles can be scaled down to create miniature bio-inspired acoustic sensors, including MEMS-based devices [18], [21], [22]. Thus, 3D printing not only facilitates current research through accessible, controllable, scaled replicas but also paves the way toward practical implementations of insect-inspired auditory mechanisms in compact sensing systems. The non-destructive nature of these methods makes them suitable for future studies of fossilised insect hearing organs through 3D printing replicas and integration of hard and soft materials. Additionally, the approach supports the 3Rs principles (Replacement, Reduction, and Refinement) by reducing dependence on live insects, minimising experimental variability, and improving reproducibility and control [23].

## 2 Methodology

### 2.1 Specimen

The specimens of *C. gorgonensis* (Tettigoniidae: Copiphorini), which are endemic to Gorgona National Natural Park, Colombia, were imported to the United Kingdom in 2015 under a research permit issued by the Colombian Authority (DTS0-G-090 14/08/2014). Following the completion of bioacoustics experiments [9], the live specimens were preserved in increasing concentrations of ethanol-filled centrifuge tubes up to 80 % (to reduce excess water loss and shrinkage) and subsequently stored at −22 °C in a dedicated freezer at the University of Lincoln for further analysis and imaging.

### 2.2 Micro-computed tomography (µCT) scanning

Two preserved adult *C. gorgonensis* specimens were imaged on a SkyScan 1172 µCT scanner (Bruker Corporation, Billerica, MA, USA), each tuned to a different structure. For the acoustic trachea, the anterior half of the first insect (head, prothorax and attached forelegs) was scanned without staining at 55 kV, 200 µA, 350 ms exposure and 0.1° rotation steps, giving a 5 µm isotropic voxel size that resolved the spiracle and acoustic trachea. The second specimen's left foreleg was excised, stained for 24 h in 1 % iodine-ethanol to enhance membrane contrast, rinsed with ethanol and desiccated overnight in hexamethyldisilazane (HMDS) to minimise tympanal deformation. The dried leg was scanned at 50 kV, 180 µA, 300 ms exposure and 0.1° rotation steps, yielding 1.5 µm isotropic voxels that resolved the tympana together with their cuticular pinnae. The raw projection data were reconstructed and exported as 16-bit TIFF orthogonal slices using NRecon software (v.1.6.9.18, Bruker Corporation, Billerica, MA, USA). The 3D-printed parts were additionally scanned at 50 kV, 180 µA, 300 ms exposure, 0.1° rotation steps and 5 µm isotropic voxels to verify print quality and dimensional accuracy.

### 2.3 Deep (AI) semantic segmentation and volumetric reconstruction

**Fig. 1:**
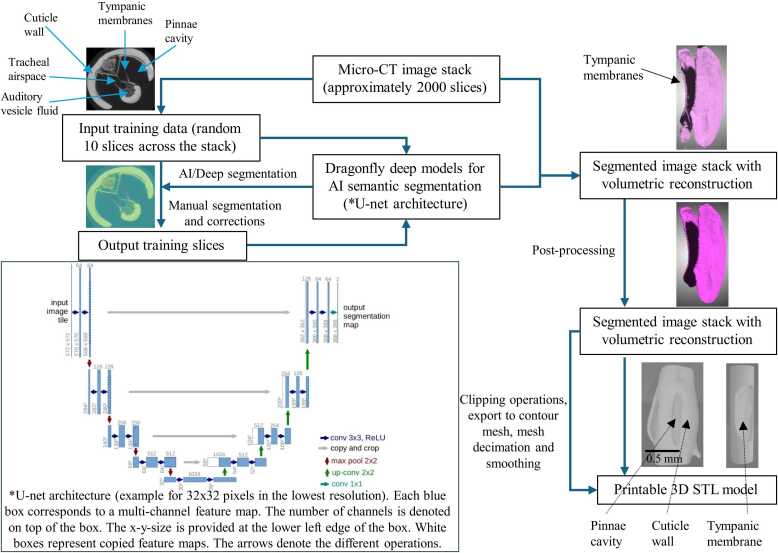
Deep learning-based semantic segmentation pipeline of the katydid outer ear for 3D reconstruction and fabrication. A U-Net convolutional neural network was trained on annotated µCT slices to segment the outer ear structures, including the cuticle, tympana, and air spaces. The model was refined over multiple iterations and applied to the full dataset to generate volumetric segmentations. These were processed to isolate the tympana and cuticle, enabling export as high-fidelity STL files for 3D printing.

Semantic segmentation of the insect outer ear was performed using the deep learning module in Dragonfly 3D software (Object Research Systems, Montreal, Canada), utilising a U-Net convolutional neural network architecture for iterative model training and refinement (Fig. 1) [24]. Semantic segmentation was employed instead of purely manual segmentation due to its significant advantages in accuracy, efficiency, and reproducibility. Manual segmentation of complex biological structures such as the tympanic membranes and acoustic trachea is extremely time-consuming and prone to user bias, as the boundaries between cuticle, air spaces, and soft tissue are often difficult to distinguish in µCT data. In contrast, deep learning-based segmentation using the U-Net architecture allows the model to learn subtle textural and intensity differences across large image datasets, enabling consistent and precise identification of anatomical features. This automated approach ensures high-fidelity reconstruction of the intricate 3D geometry of the ear, capturing realistic anatomical variations rather than relying on idealised or oversimplified models, which is essential for producing accurate biophysical and acoustic simulations.

For tympanic membrane segmentation, approximately 2000 high-resolution µCT slices of the insect’s outer ear were imported into the software, from which ten slices were randomly selected to create a representative training subset, referred to as *Input Slice V1*. These slices were manually segmented to define multiple regions of interest (ROIs), including the cuticle, tympana, and air spaces, producing the corresponding labelled dataset, *Output Slice V1*. This dataset served as the ground truth for initial model training.

The U-Net model was first trained using *Input Slice V1* and *Output Slice V1*. To evaluate and improve model accuracy, a new set of ten slices (*Input Slice V2*) was randomly extracted from the full dataset and segmented using the trained model. The output segmentations (*Output Slice V2*) were manually reviewed and corrected to rectify classification errors. These corrected slices were then used to retrain the model. This process of prediction, correction, and retraining was repeated through three further iterations (up to *Input/Output Slice V5*), progressively enhancing model performance. After four training cycles, the model achieved accurate classification of anatomical structures with minimal need for manual correction and was applied to segment the entire volumetric dataset automatically.

After the segmentation of the outer ear, the tympana were isolated from the surrounding cuticle to enable material-specific 3D printing. A virtual cylindrical tool was precisely aligned with the tympanal region in the segmented volume to define the separation boundary. A clipping operation was performed to partition the flexible tympanal membranes from the rigid cuticle structures. This operation did not alter the true anatomical boundary of the tympanic membranes, as the clipping tool only separated the membranes and cuticle along the existing segmentation mask without modifying the underlying geometry. This operation generated two physically distinct models for printing: a flexible region composed mostly of tympanum and a rigid supporting cuticle. These were later assembled into a single anatomical model, faithfully replicating the geometry and functional differentiation of the insect’s outer ear.

A similar deep learning approach was employed to segment the acoustic tracheal system, using approximately 1500 µCT slices. The U-Net model was trained to distinguish the air-filled tracheal lumen from surrounding tissues, enabling accurate reconstruction of the acoustic trachea.

**Fig. 2:**
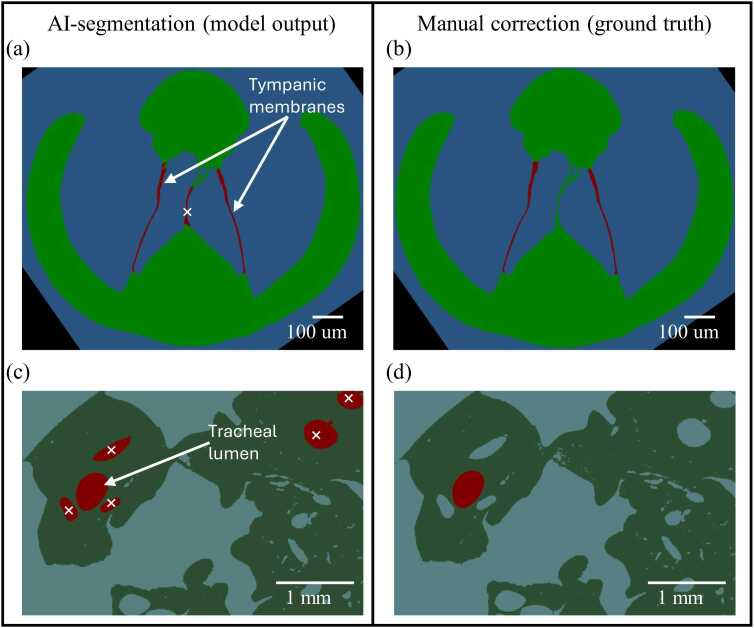
Validation of µCT segmentation for the outer-ear tympanic membranes and the hollow lumen of the acoustic trachea. Left panels show predictions from the trained Dragonfly deep segmentation models, and right panels show manual segmentation (ground truth). False-positive regions are marked with white crosses. For the outer-ear segmentation, occasional false positives occurred along the medial septum, indicating a tendency of the model to prioritise membrane thickness. For the tracheal lumen, false positives corresponded to other enclosed air-filled structures within the head capsule, reflecting the model’s sensitivity to internal air spaces. These errors were corrected manually during post-processing.

Segmentation accuracy was assessed by comparing the AI-segmented outputs with manually corrected ground truth on a subset of representative slices for both the outer-ear tympanic membranes and the tracheal lumen. After four iterative cycles of correction and retraining, the model generalised reliably across the remaining µCT volume and required only minor post-processing during final inspection. A qualitative comparison is provided in Fig. 2, with typical false-positive regions marked for clarity. Quantitative performance was evaluated using Dice coefficients calculated from ten manually segmented slices per class taken uniformly across the scan, and these values are presented in Table 1.

**Table 1:** Segmentation performance for each anatomical class, expressed as Dice coefficients and IoU values. Dice was calculated from ten manually segmented slices per structure taken uniformly across the µCT volume, and IoU values were derived directly from the Dice coefficients.

Segmentation target	Anatomical class			
Outer ear	Tympanic membranes	Rest of the body	Background	Overall
Dice	0.743	0.906	0.969	0.953
IoU	0.593	0.825	0.941	0.907
Acoustic trachea	Tracheal lumen	Rest of the body	Background	Overall
Dice	0.761	0.998	0.990	0.987
IoU	0.612	0.996	0.980	0.973

The segmented structures were converted to contour meshes in Dragonfly 3D. The trachea was offset by 10 µm for wall thickness to preserve the hollow lumen dimensions. Mesh decimation was applied to reduce file size (<150,000 triangles), followed by two iterations of Laplacian surface-preserving smoothing. Quantitative assessments confirmed that these steps did not meaningfully alter biologically relevant geometrical features, with changes in tympanic membrane thickness and tracheal lumen diameter remaining below 1 %.

### 2.4 3D printing of segmented structures

Studies on the mechanical properties of insect cuticles have shown that their stiffness varies widely, with soft cuticle ranging from approximately 1 kPa to 50 MPa, while sclerotised (hardened) cuticle can reach values between 1 GPa and 20 GPa [25]. Based on this, two printing materials were selected: polylactic acid (PLA) and thermoplastic polyurethane (TPU), manufactured by Bambu Lab and procured from AdditiveX. PLA, with a Young’s modulus of about 2.7 GPa, was used to represent the rigid cuticular structures of the outer ear, providing the necessary stiffness and structural stability. In contrast, TPU (TPU 95AH), with a Young’s modulus of around 9.8 MPa, was chosen to replicate the flexible tympanic membranes, allowing realistic vibration and deformation under sound excitation similar to the natural tympanum. Additionally, models with opposite material combinations (PLA tympana and TPU cuticle) were also printed to compare how differences in material stiffness influence the overall acoustic and mechanical behaviour of the structure. PLA was also selected for the fabrication of the scaled acoustic trachea, as atomic force microscopy (AFM) nanoindentation measurements of the native tracheal wall revealed a broad distribution of elastic moduli, with a mean of 5200 MPa and a median of 1030 MPa, indicating a predominantly stiff structural profile [26].

**Fig. 3:**
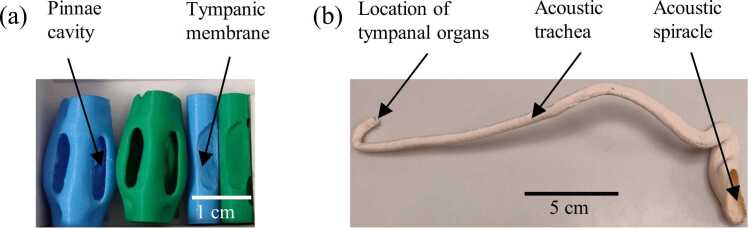
3D-printed models of the katydid outer ear and acoustic trachea. (a) 15-times-scaled outer ear components printed using FDM and SLA methods. PLA (green) and TPU (blue) structures were fabricated separately and assembled for acoustic testing. (b) 20-times-scaled acoustic trachea printed in PLA using FDM. Only the outer wall was printed to preserve the hollow lumen, replicating the native tubular geometry essential for acoustic functions.

The outer ear and acoustic trachea were 3D-printed using the fused deposition modelling (FDM) process on a Bambu P1S printer (Bambu Lab, Shenzhen, China) operated via Bambu Studio software (Version 1.9.7.50). The katydid outer ear model was enlarged 15 times, while the acoustic trachea was enlarged 20 times to fabricate scaled physical replicas suitable for acoustic experimentation (Fig. 3). The default printer settings, based on the drop-down material profiles in Bambu Studio, were used for most parameters. A few key settings were adjusted to improve print quality and structural accuracy: a 0.2 mm nozzle diameter, 0.06 mm layer height, nozzle temperature of 230 °C, and bed temperature of 30 °C were applied for both PLA and TPU structures. For the outer ear models, a 100 % infill with concentric layering was used to ensure full rigidity, whereas the acoustic trachea was printed without infill, using a 0.25 mm shell thickness to maintain the hollow internal cavity essential for acoustic functionality.

For each configuration (outer-ear model and tracheal model), two to three prints were produced. These models were inspected visually and using µCT to assess print quality, including tympanic membrane thickness, tracheal lumen diameter, surface porosity and the absence of defects or wear. From this set, the model with the best print quality was selected for measurement.

### 2.5 Laser-doppler vibrometry (LDV) on scaled tympana of the outer ear model

**Fig. 4:**
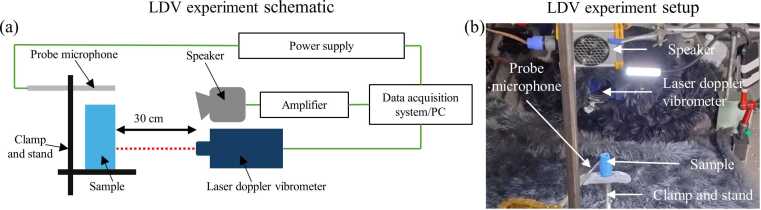
Laser-Doppler vibrometry (LDV) setup for vibration analysis of 3D-printed insect outer ear models. (a) Schematic of the experimental setup showing a speaker delivering sound stimuli to the sample, with vibration velocity measured by LDV and sound pressure recorded by a probe microphone. (b) Experimental configuration highlighting key components, including the speaker, LDV head, probe microphone, and 3D-printed sample mounted on a clamp and stand.

LDV experiments were conducted in an acoustic chamber designed to minimise external noise and vibrations (Fig. 4). All equipment and samples were placed on a vibration isolation air table to reduce measurement noise. The samples were mounted on a beam supported by a magnetic stand and fixed in position using adhesive putty. The orientation of the samples was adjusted to focus the laser beam from the scanning vibrometer (Polytec PSV-500 scanning head; Waldbronn, Germany) onto the tympanic membrane.

Avisoft ultrasonic speaker Vifa with SPEAKON connector (Avisoft-UltraSoundGate; Glienicke/Nordbahn, Germany), positioned 30 cm from the samples and secured on a magnetic stand, was used to deliver acoustic stimuli. The loudspeaker was connected to a portable ultrasonic power amplifier (Avisoft-UltraSoundGate; Glienicke/Nordbahn, Germany) to ensure controlled sound delivery. A 1/8″ precision pressure microphone (Bruel & Kjaer, 4138; Nærum, Denmark) was placed adjacent to the samples, facing the speaker and connected to a power supply (GRAS 12AA 2-Channel Power Module with gain and filters; Holte, Denmark). The microphone was calibrated using a B&K Type 4237 sound pressure calibrator (94 dB at 1 kHz) to ensure accurate sound pressure measurements.

The scanning vibrometer, loudspeaker, and microphone were connected to a PSV-500 internal data acquisition board (Polytec PSV-500 vibrometer front-head; Waldbronn, Germany), which interfaced with a computer running Polytec 10.1.1 software for system control and data analysis. A broadband stimulus consisting of periodic chirps with a simulated frequency range of 2–10 kHz was delivered by the loudspeaker. To ensure consistent stimulus intensity, the amplitude of the broadband signal was mathematically corrected within the software, providing a uniform sound pressure level of 60 dB across all frequencies.

During measurements, the tympanic membrane region was selected within the software, and the laser focus was systematically adjusted to ensure high-quality data acquisition. Each tympanic membrane was scanned using 420 measurement points, uniformly distributed across the membrane surface, and no retro-reflective coating was required, as the printed membrane surfaces provided sufficient optical reflectance for reliable scan. The vibrations of the tympanic membrane were recorded in the frequency domain at a sampling frequency of 512 kHz. The acquisition software applied a built-in five-point spectral averaging routine, and the resulting spectra were visually checked to confirm that no variability was apparent across the software-averaged sweeps.

Data were post-processed and analysed using Polytec 10.1.1 software, and the final outputs were exported and visualised using MATLAB libraries for graph plotting and detailed analysis. Resonance peaks and corresponding gains were extracted in MATLAB by identifying local maxima in the displacement magnitude spectrum using a peak-finding algorithm, and bandwidths were estimated from the continuous frequency interval within which the response remained within 3 dB of each peak. Because only one selected print per configuration was used, and the acquisition software returned only averaged spectra, no confidence intervals or error bars were computed. Instead, spectral stability was confirmed by visual inspection of successive software-averaged sweeps.

### 2.6 Acoustic experiment on scaled acoustic trachea

The 20 × scaled acoustic trachea was fixed in place using a clamp and stand system, with the model secured gently via a plastic clip to minimise mechanical interference. Acoustic measurements were conducted in a sound-isolated acoustic chamber using a combination of free-field and near-field stimuli.

A 1/8″ precision pressure microphone (Bruel & Kjaer, 4138; Nærum, Denmark), connected to a conditioning amplifier, was positioned adjacent to the spiracle opening to monitor and calibrate reference sound pressure levels during each trial. Two 25-mm probe microphones (Type 4182, Brüel & Kjær, Nærum, Denmark), each with a 25-mm-long and 1.24-mm inner diameter probe tube, were connected to preamplifiers (Type 2633, Brüel & Kjær). One probe was inserted into the spiracle opening, and the other at the distal end of the tracheal lumen, allowing simultaneous measurement of input and output sound pressures. All microphones were calibrated before testing using a B&K Type 4237 sound pressure calibrator (94 dB at 1 kHz) to ensure accurate and reproducible measurements.

**Fig. 5:**
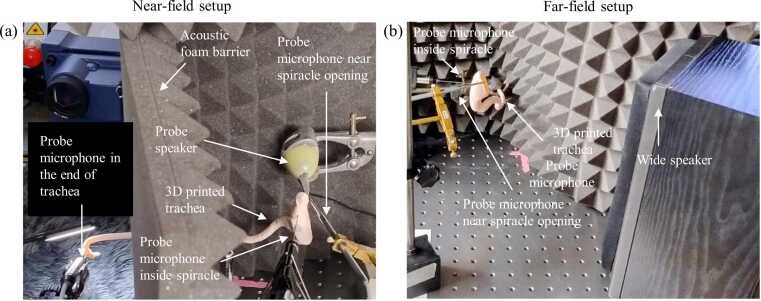
Acoustic characterisation setups for 3D-printed scaled katydid trachea. (a) Near-field configuration using a custom probe speaker positioned 1 cm from the spiracle to deliver focused acoustic input. Sound pressure was recorded using three probe microphones placed near the spiracle opening, inside the spiracle, and at the distal end of the trachea. An acoustic foam barrier was positioned to minimise cross-interference between the input and output ends. (b) Far-field configuration using a wide speaker placed 30 cm from the spiracle to simulate distant sound sources. Probe microphones recorded input and internal pressures, enabling comparison of acoustic transmission under different spatial stimulation regimes.

Two acoustic stimulation conditions were tested. In the near-field configuration (Fig. 5a), a custom probe speaker was positioned approximately 1 cm from the spiracle. This speaker was assembled by encasing an earbud in acoustic foam and attaching a plastic conical nozzle to direct the output into the spiracle opening, ensuring localised delivery of sound pressure. In the far-field configuration (Fig. 5b), a broadband loudspeaker (wide speaker) was placed 30 cm away from the spiracle and oriented directly towards it. This setup simulates distant sound sources, where pressure waves arrive as planar fronts with relatively uniform phase and amplitude. Both speakers were connected to the Avisoft-UltraSoundGate power amplifier, which was connected to a PSV-500 internal data acquisition board for post-processing results using the Polytec 10.1.1 software.

For both stimulation conditions, a broadband signal from 0.5 to 4 kHz was delivered at a flattened 40 dB SPL. To acoustically isolate the ends of the printed trachea and prevent airborne coupling to the distal microphone, multiple pyramid-foam panels (44 × 44 cm, pyramid height 5 cm) were positioned between the spiracle and the distal opening. Additional foam panels were placed around the loudspeaker and spiracle to minimise lateral radiation and ensure that only the spiracle received the forward-propagating stimulus. Control measurements with the distal probe microphone positioned outside the tracheal lumen recorded negligible sound pressure, confirming that the distal measurements reflect transmission through the lumen rather than airborne excitation. The 25-mm B&K probe microphones operate as pressure receivers within their narrow probe tubes, which substantially shield the diaphragm from off-axis sound, ensuring that only pressure waves propagating along the lumen contribute to the recorded signal. The probe microphones were inserted only to the inner edge of the tracheal wall, avoiding any contact with the printed structure to prevent any alteration of the internal acoustic field or the introduction of standing-wave artefacts.

Tracheal recordings were processed using the same acquisition and five-point spectral averaging as the LDV measurements. Repeated re-entries of microphone produced identical peaks, gains and bandwidths, so a single final dataset was post-processed in MATLAB using the same peak-finding approach applied to the distal-to-spiracle pressure ratio, and phase responses were obtained by unwrapping the complex pressure ratio across the stimulus band and checking that the resulting curves remained continuous without unintended ±360° jumps.

## 3 Results

### 3.1 Displacement profile of real insect vs 3D-printed TPU tympanic membrane

**Fig. 6:**
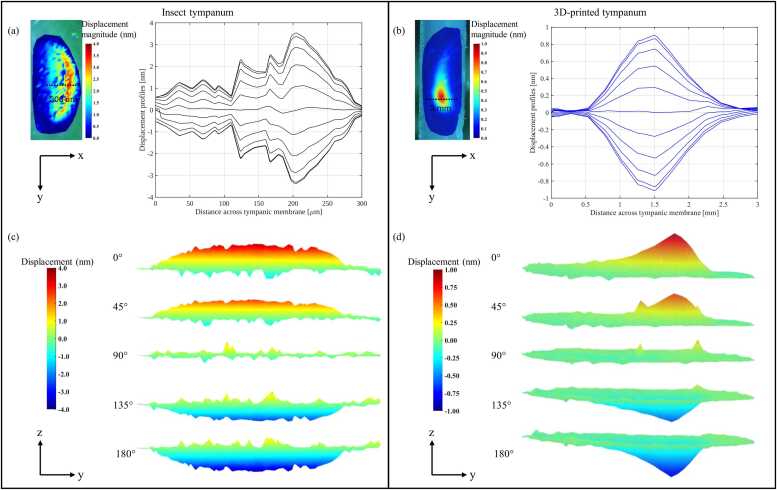
Displacement profiles of the insect and the 3D-printed isolated tympanic membrane. (a) LDV map of a live *C. gorgonensis* tympanum at 10 kHz after pinna removal and the corresponding displacement profiles at different phases sampled along the dashed 300 µm transect. (b) LDV map of the 15-times-scaled TPU membrane at 7 kHz with displacement profiles at different phases taken along the dashed 3 mm transect. (c) Sequential surface plots (five equally spaced phase snapshots within one stimulus cycle) of an *in-vivo C. gorgonensis* tympanum with the pinna removed (10 kHz excitation). (d) Corresponding phase snapshots of the 15-times-scaled TPU membrane driven at 7 kHz.

The displacement fields of a live *C. gorgonensis* tympanum, with its pinna surgically removed and stimulated at 10 kHz from a previous LDV study [9] and of the isolated 15-fold scaled TPU membrane driven at 7 kHz are displayed in Fig. 6. Spatial line profiles extracted across the tympana for both cases show negligible motion at the edges. Moving towards the centre, displacement rises steeply to a broad crest and then decays slightly asymmetrically for the real insect tympana toward the distal edge (Fig. 6a) and symmetrically for the 3D-printed tympana (Fig. 6b). The living membrane reaches a peak of around 3.6 nm at roughly 200 µm from the proximal attachment, whereas the printed membrane peaks at around 0.9 nm at roughly 1.5 mm from the attachment.

Five snapshots taken at equal phase intervals within a single acoustic cycle reveal that the live *C. gorgonensis* tympanum (pinna removed, 10 kHz stimulus) develops a broad, dome-shaped crest spanning across the membrane, with peak excursions of about ±4 nm, as presented in Fig. 6c. The complementary sequence for the 15-times larger TPU print (7 kHz; Fig. 6d) reveals a much narrower zone of activity: a sharp central ridge rises and falls while most of the surrounding film remains close to baseline, and the displacement range is limited to approximately + 1 nm to –1 nm.

**Fig. 7:**
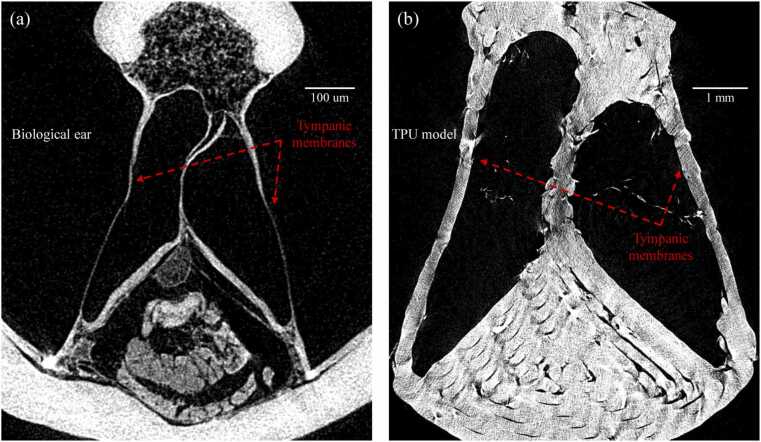
Cross-sections of the biological and printed tympanic membranes. (a) µCT slice through the biological ear showing the native tympanic membranes with strongly graded thickness. (b) Corresponding slice through the TPU outer-ear model, in which the printed tympanic membranes have approximately uniform thickness set by the minimum printable layer height. Mean membrane thickness in the insect was 8.04 ± 3.62 µm, whereas the scaled TPU membranes were 265.23 ± 38.55 µm (equivalent to 17.68 ± 2.57 µm at 15 × down-scaling), illustrating both the loss of thickness grading and the limits of fabrication accuracy.

The µCT cross-sections of the biological tympanum and the scaled TPU replica are shown in Fig. 7. The native membrane exhibits significant variation in thickness across its cross-section, with a mean value of 8.04 ± 3.62 µm. In contrast, the printed TPU membrane forms a uniform measured thickness of 265.23 ± 38.55 µm, which corresponds to an equivalent biological thickness of 17.68 ± 2.57 µm when scaled down 15-fold.

### 3.2 Laser-doppler vibrometry of 3D-printed assembled outer-ear structures

**Fig. 8:**
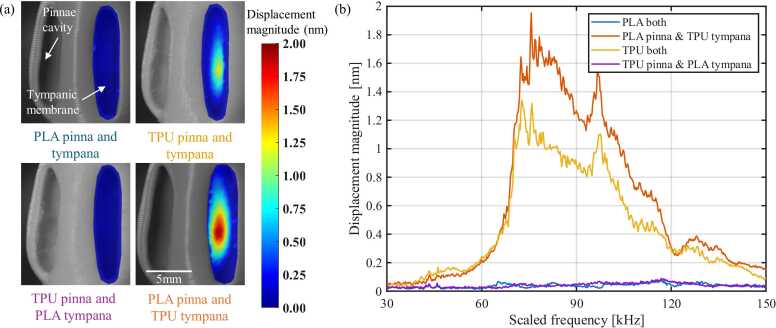
Pinna-membrane material pairing modulates tympanic vibration in assembled outer ears. (a) Displacement maps at a scaled 80 kHz for four pinna/membrane combinations. (b) Displacement magnitude at the membrane centre versus scaled frequency (30–150 kHz). The PLA-pinna/TPU-membrane assembly peaks at 1.93 nm.

Complete pinna-tympanic membrane assemblies were fabricated in four material pairings: both PLA pinnae and membrane, both TPU pinnae and membrane, TPU pinnae with PLA membrane and PLA pinnae with TPU membrane (Fig. 8a). As the 3D-printed outer-ear models were 15 times larger than the actual insect, the acoustic excitation was conducted in the 2–10 kHz range, corresponding to 30–150 kHz in the natural scale of bat echolocation calls. The recorded data were subsequently post-processed and frequency-scaled (Fig. 8b) for direct comparison of *C. gorgonensis* in vivo measurements [10]. The results revealed that the assembly that employed the flexible TPU for the tympanic membrane exhibited substantial motion, displaying a broad resonance between 70–110 kHz. The PLA-pinna/TPU-membrane model produced a single displacement lobe with a peak amplitude of 1.93 nm at 75 kHz, whereas the all-TPU construct reached 1.27 nm at the same frequency. Both PLA-membrane combinations remained below 0.10 nm and showed no coherent spatial pattern.

**Fig. 9:**
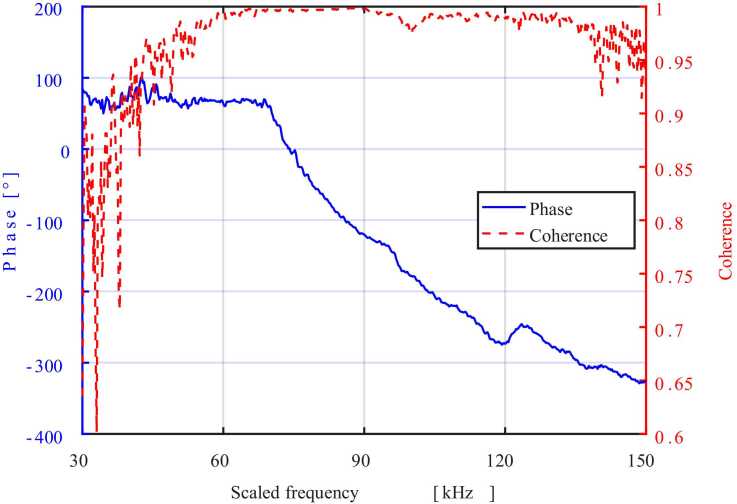
Phase (blue) and transfer-function coherence (red) between the acoustic stimulus and membrane displacement across 30–150 kHz. Coherence exceeds 0.95 over most of the band, while phase decreases smoothly by ∼400°, indicating single-mode propagation.

For the PLA-pinna/TPU-membrane construct, the transfer-function coherence between the acoustic stimulus and membrane displacement exceeded 0.95 from 60 kHz to ∼140 kHz and remained above 0.90 across the measured 30–150 kHz range (Fig. 9, red dashed line). The associated phase response (blue line) declined smoothly by ∼400° after 70 kHz, without abrupt jumps, indicating a largely linear, single-mode membrane motion. The high coherence confirms that most of the incident acoustic energy reached and drove the membrane throughout the frequency band examined.

### 3.3 Sound transmission through the 3D-printed acoustic trachea

**Fig. 10:**
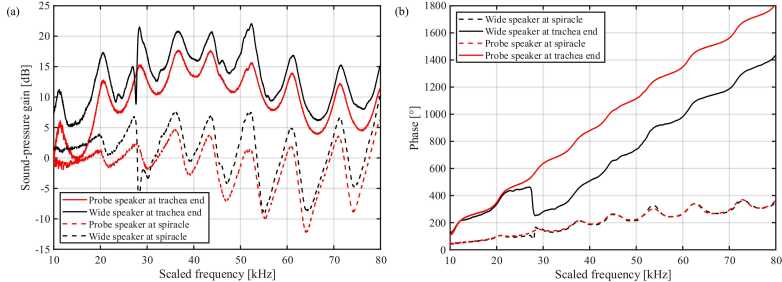
Sound-pressure gain and phase along the 3D-printed acoustic trachea. (a) Sound-pressure gain measured at the spiracle (dashed traces) and at the distal terminus (solid traces) for a near-field probe source positioned 3 mm from the spiracle (red) and for a far-field loudspeaker 30 cm away (black). Peaks of 17–21 dB at the terminus correspond to troughs at the spiracle, indicating that the opening acts as a frequency-selective filter while the exponential canal amplifies the transmitted bands. (b) Unwrapped phase for the same four conditions. Phase accumulates smoothly to ∼1800° over the scaled 10–80 kHz sweep at the trachea end, confirming single-mode propagation along the horn; stimulus geometry alters phase gradient by < 10 %.

The 20-times-scaled trachea, printed in PLA, was excited with broadband stimuli delivered either by a miniature probe speaker positioned 3 mm from the spiracle (“probe” condition) or by a 100 mm loudspeaker (wide speaker) placed 30 cm away (far-field condition) at 40 dB sound pressure level. As the tracheal model was enlarged 20 times, the acoustic experiments were conducted between 0.5–4 kHz, equivalent to 10–80 kHz in the actual insect. This band captures the full frequency range in which the acoustic trachea functions *in vivo*, where its dominant resonance and maximum gain occur around 20–30 kHz and do not extend into the high-frequency bat-detection range [9]. Above ∼80 kHz, sound reception is governed primarily by the pinnae rather than the trachea. The chosen stimulus band, therefore, reflects the trachea’s biologically relevant operating range and aligns with the frequency span used in numerical simulations of the native structure [16]. Pressure gain and unwrapped phase change were recorded at the spiracle opening and at the distal (tibial) end of the tube, and the results are shown in Fig. 10.

Under far-field excitation (Fig. 10a, black curves), the tracheal end exhibited a series of broad gain maxima at ∼22, 32, 42, 52, 62 and 72 kHz, reaching 17–21 dB. At the spiracle, gain remained near 0 dB below 20 kHz and oscillated between −5 and +7 dB above that range. The near-field excitation (probe stimulus, red curves) produced the same spectral pattern but at slightly lower amplitude: peaks of 12–16 dB at the terminus and −6 to +5 dB at the spiracle. Thus, in both speaker configurations, the trachea increased sound pressure by more than an order of magnitude at its distal end across multiple ultrasonic bands.

The corresponding phase response (Fig. 10b) rose approximately linearly with frequency at the tracheal terminus, accumulating ∼1800° between 10–80 kHz for the probe stimulus (red solid line) and ∼1400° for the far-field stimulus (black solid line). The phase measured at the spiracle (dashed lines) remained below 350° over the same band, and no abrupt discontinuities were observed, indicating a smooth propagation of the dominant acoustic mode along the tube. These measurements show that the printed horn preserves a frequency-dependent pressure build-up along its length while maintaining high coherence between input and output across the 10–80 kHz sweep.

### 3.4 Comparison with published results

**Fig. 11:**
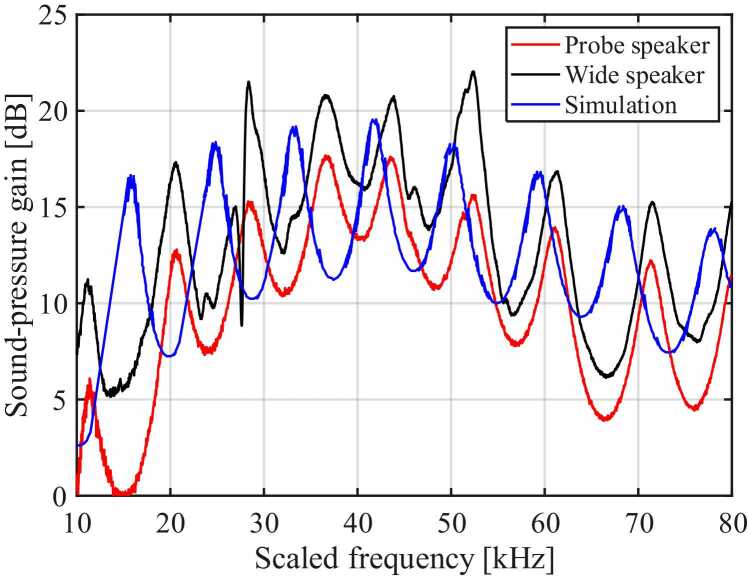
Sound-pressure gain at the tracheal terminus measured with probe (red) and wide (black) sources compared with the finite-element prediction (blue) over a scaled 10–80 kHz range; peaks align within ±2 kHz and ±3 dB. Printed tracheal gain closely matches finite-element simulation.

Superimposing the measured sound-pressure gains onto the finite-element study [16] shows peaks aligning within ±2 kHz and amplitudes within ±3 dB across 10–80 kHz (Fig. 11). The main acoustic features of the 3D-printed outer ear and trachea models are summarised in Table 2.

**Table 2:** Comparison of key acoustic features measured *in vivo* and in simulation with those obtained from the scaled 3D-printed replicas. Values for the printed models are from the present measurements, and the *in vivo* and simulation values are taken from published studies of *C. gorgonensis* (references in brackets).

Features	Actual insect (*in vivo*)	3D-printed (scaled model)	Simulation
Tympana thickness	8.04 ± 3.62 µm [9]	265.23 ± 38.55 µm (17.68 ± 2.57 µm, 15 × scaled down)	---
Isolated Tympana max displacement	±4 nm [9]	±1 nm	---
Tympana resonance bandwidth with cavity	∼80–120 kHz [10]	∼70–110 kHz, LDV ∼60–120 kHz, microphone [10]	∼50–120 kHz, smooth changes [10]
Acoustic trachea sound pressure gain	∼+ 15 dB [9]	∼+ 21 dB	∼+ 20 dB [16]
Acoustic trachea phase shift at 23 kHz	460–490° (∼1.3–1.4 cycles) [9]	460° (∼1.3 cycles)	---

## 4 Discussion

LDV revealed that the TPU film produced a single-lobe vibration pattern comparable to that of the living *C. gorgonensis* exposed tympanum (see Supplementary Videos 1 and 2). Over a full acoustic cycle, both the native and the printed membranes rise on one side and settle back towards the edge (Fig. 6). However, the overall displacement patterns differ markedly. The biological tympanum spreads its motion over a broad region and reaches amplitudes of approximately ±4 nm, whereas the TPU replica produces a sharper, more localised ridge with a reduced amplitude of about ±1 nm. These differences arise primarily from fabrication constraints rather than segmentation error. Although the tympanal geometry was captured accurately in the 3D model, the printed TPU membrane cannot reproduce the thin, spatially graded and mechanically heterogeneous structure of the native ear. The printing resolution imposes a minimum line width of around 250 µm, which at 15 × scale gives an effective biological thickness of roughly 18 µm, approximately twice the mean thickness of the insect tympanum and several times greater than its thinnest regions (Fig. 7). The printed membrane is therefore relatively uniform in thickness and was assembled without controlled pre-tension, whereas the natural tympanum exhibits strong thickness gradients, heterogeneous stiffness and inherent pre-strain. These factors lead to a more concentrated, high-curvature deflection in the TPU membrane, while the native tympanum displays broader motion and resonates most strongly near 7 kHz, compared with 10 kHz in the enlarged model due to differences in material properties. Crucially, these discrepancies influence only the local modal behaviour of the membrane and do not affect the pinna-driven cavity resonance, which is governed predominantly by geometry rather than by the fine mechanical properties or natural frequency of the tympanal tissue.

Supplementary material related to this article can be found online at doi:10.1016/j.csbj.2025.12.003.

The following is the Supplementary material related to this article Video S1.

Video S1

The following is the Supplementary material related to this article Video S2.

Video S2

LDV on assembled structures (Fig. 8) showed that structures comprising TPU membranes resonate between 70 and 110 kHz, closely aligning with the insect’s native bat-detection band (see supplementary video 3). This confirms that katydid pinnae behave as mechanical amplifiers for selective ultrasonic bands, a function previously inferred from LDV on intact legs [10]. The PLA pinna cavity with TPU membrane produced a 1.93 nm peak, 50 % larger than the all-TPU construct. This confirms that the acoustic properties of these structures are influenced more by their architecture than by material properties such as stiffness. Still, the PLA/TPU pairing captures some biological impedance contrast, as the pinnae fabricated with PLA are orders of magnitude stiffer than TPU, reflecting most sound waves onto the tympana [25]. The higher stiffness PLA membrane thus showed no sign of resonance. These findings are consistent with previous LDV data from real-ear measurements (Table 2), which showed increased tympanic membrane vibration above 80 kHz in response to a broadband stimulus (20–120 kHz), with a resonant peak around 107–111 kHz attributed to pinnae-driven amplification [10]. Phase fell gradually across the measured band, indicating essentially direct pressure loading with no abrupt modal transitions. The assembled PLA-pinna/TPU-membrane model exhibited coherence > 0.95 throughout the frequency band (Fig. 9), confirming that nearly all incident acoustic energy was transmitted to the membrane.

Supplementary material related to this article can be found online at doi:10.1016/j.csbj.2025.12.003.

The following is the Supplementary material related to this article Video S3.

Video S3

Pressure mapping along the 20-times-scaled PLA acoustic trachea revealed that gain at the spiracle oscillates between −5 dB and + 7 dB, whereas gain at the distal tip reaches 17–21 dB (Fig. 10a). The oscillating sound-pressure gain indicates that the spiracle operates as a frequency-selective filter, while the exponential canal functions as a passive amplifier [27]. Phase advanced smoothly to ∼1800° over 10–80 kHz (Fig. 10b), matching a similar profile measured in intact insect trachea. The phase shift at 23 kHz was approximately 460° (around 1.3 cycles), the same as the real insect tracheal phase shift (460–490° or 1.3–1.4 cycles) [9], supporting the consistency of the 3D-printed model for experimentation and analysis (Table 2). Changing from a distant far-field loudspeaker to a near-field probe altered peak gain by < 5 dB and phase by < 10 %, suggesting that the horn’s filtering properties are largely source-independent. This agreement supports the 3D-printed scale model, providing physical confirmation of the predicted acoustic behaviour. Thus, the printed horn can be instrumented at arbitrary points and offers a powerful benchmark for refining future simulations.

As this work presents the first complete scaled physical realisation of both the outer ear and acoustic trachea of *C. gorgonensis*, only one selected print per configuration was examined in detail. While spectral stability across software-averaged sweeps was confirmed, across-print variability was not quantified and should be addressed in future work. Nevertheless, the aim of this study was to establish the feasibility and acoustic behaviour of accurately reproduced geometries, and the selected prints provided stable and interpretable measurements suitable for this purpose.

The passive mechanical behaviour demonstrated by the scaled pinnae and acoustic trachea is also relevant from an engineering perspective. Many biological hearing systems rely on intrinsic mechanical preprocessing, such as frequency-dependent phase accumulation, geometric filtering and impedance contrast, to shape acoustic input before neural transduction [28]. These geometry-driven transformations operate without active power input, offering principles that could inform low-energy, directionally sensitive miniature sensors. The µCT-segmentation-printing workflow developed here similarly aligns with emerging computational-to-fabrication pipelines described in recent bio-fabrication literature, where imaging-driven modelling and digital design enable rapid prototyping of biomimetic structures [29]. Such integration underscores how validated structure-function relationships from insect ears can support future development of bio-inspired MEMS microphones and mechanically efficient acoustic sensors.

## 5 Conclusion

The scaled 3D-printed models of the *C. gorgonensis* outer ear reproduced key acoustic features of the biological hearing system. The combination of flexible membranes and rigid pinnae acted as a physical analogue of the insect’s bat-detection mechanism, showing mechanical gain within the ultrasonic echolocation band of bat calls. Despite the mismatch in the membrane mechanics (due to stiffness mismatch, scaling constraint and printing resolution), the assembled outer-ear structures reproduce the correct ultrasonic resonance band (70–110 kHz) observed in vivo. This is because cavity-driven amplification by the pinnae depends primarily on geometric acoustic interactions (cavity volume, opening size, and path lengths) rather than the fine modal structure of the tympanal tissue. Also, the printed acoustic trachea exhibited the filtering and amplification behaviour previously predicted only by numerical simulations, confirming that the exponential horn acts as a passive amplifier and the spiracle as a filter for frequency-selective input. Together, these results show that although the printed membranes do not reproduce native mechanical properties, the high-fidelity geometric reconstruction enables fabrication of both the outer-ear and tracheal pathways with good acoustic similarity to a real insect.

The process of producing scaled physical models provides a reliable experimental platform for testing hypotheses about miniature pressure-difference receivers while reducing the need for live specimens. The controlled, repeatable 3D-printing process, with its design freedom, allows future exploration of other key parameters such as the influence of stiffness, cavity geometry and horn shape. In addition, the non-destructive workflow described may be extended to reconstruct replicas of fossilised insect hearing organs, incorporating both soft and hard materials based on tissue types through multi-material 3D printing, to explore their acoustic performance and evolutionary significance. This provides a powerful tool for acoustic biophysics while supporting the 3Rs principles by reducing dependence on live insects. Importantly, this interdisciplinary approach demonstrates that insect-inspired hearing mechanisms have the potential to be translated into scalable miniature engineering systems.

The validated relationships between cavity and trachea structure, resonance, and phase delay provide a foundation for future development of miniaturised mechanical adapters, MEMS-based microphones and bio-inspired directional sensors that exploit passive acoustic filtering and amplification, reducing dependence on electronic gain and software post-processing and thereby improving energy efficiency. As 3D printing technology advances, with higher resolution and more precise multi-material integration, replicating these biological structures at their actual scale may become feasible, creating opportunities to explore engineered filters and amplifiers. Future work integrating the outer-ear and acoustic trachea models into a single structure will enable direct investigation of the katydid’s dual-input hearing mechanism, providing deeper insight into its acoustic properties and further bridging biological understanding with technological innovation through tangible, nature-inspired design.

## Author statement

We confirm that this revised manuscript is original and has not been published or submitted elsewhere. All authors have approved the revised version and agree with its submission to *CSBJ*. All reviewer comments have been fully addressed, and the manuscript has been updated accordingly. The authors declare no conflicts of interest.

## CRediT authorship contribution statement

**Md Niamul Islam:** Writing – review & editing, Writing – original draft, Visualization, Validation, Software, Methodology, Investigation, Formal analysis, Data curation, Conceptualization. **Fabio A. Sarria-S:** Writing – review & editing, Visualization, Validation, Software, Methodology, Investigation, Data curation. **Fernando Montealegre-Z:** Supervision, Resources, Project administration, Funding acquisition, Conceptualization.

## Declaration of Competing Interest

The authors declare no conflict of interest.
